# Novel Protozoans in Austria Revealed through the Use of Dogs as Sentinels for Ticks and Tick-Borne Pathogens

**DOI:** 10.3390/microorganisms9071392

**Published:** 2021-06-28

**Authors:** Michiel Wijnveld, Anna-Margarita Schötta, Theresa Stelzer, Georg Duscher, Michael Leschnik, Hannes Stockinger, Per-Eric Lindgren, Gerold Stanek

**Affiliations:** 1 Institute for Hygiene and Applied Immunology, Center for Pathophysiology, Infectiology and Immunology, Medical University of Vienna, Kinderspitalgasse 15, 1090 Vienna, Austria; anna-margarita.schoetta@meduniwien.ac.at (A.-M.S.); theresa.stelzer@meduniwien.ac.at (T.S.); hannes.stockinger@meduniwien.ac.at (H.S.); gerold.stanek@meduniwien.ac.at (G.S.); 2 Department of Pathobiology, Institute of Parasitology, University of Veterinary Medicine Vienna, Veterinärplatz 1, 1210 Vienna, Austria; georg.duscher@ages.at; 3 AGES-Austrian Agency for Health and Food Safety, Spargelfeldstrasse 191, 1220 Vienna, Austria; 4 Small Animal Clinic, Department for Companion Animals, University of Veterinary Medicine Vienna, Veterinärplatz 1, 1210 Vienna, Austria; michael.leschnik@vetmeduni.ac.at; 5 Division of Inflammation and Infection, Department of Biomedical and Clinical Sciences, Linköping University, SE-581 85 Linköping, Sweden; per-eric.lindgren@liu.se; 6 Division of Clinical Microbiology, Laboratory Medicine, Region Jönköping County, SE-551 85 Jönköping, Sweden

**Keywords:** *Anaplasma*, *Babesia*, *Borrelia*, Neoehrlichia, *Rickettsia*, *Theileria*, Austria, dogs, *Ixodes ricinus*, *Dermacentor reticulatus*, *Haemaphysalis concinna*

## Abstract

We previously isolated and cultivated the novel *Rickettsia raoultii* strain Jongejan. This prompted us to ask whether this strain is unique or more widely present in Austria. To assess this issue, we retrospectively screened ticks collected from dogs in 2008. Of these collected ticks, we randomly selected 75 (47 females and 28 males) *Dermacentor reticulatus*, 44 (21 females, 7 males, and 16 nymphs) *Haemaphysalis concinna*, and 55 (52 females and 3 males) ticks of the *Ixodes ricinus* complex. Subsequently, these ticks were individually screened for the presence of tick-borne pathogens using the reverse line blot hybridization assay. In our current study, we detected DNA from the following microbes in *D. reticulatus*: *Anaplasma phagocytophilum*, *Borrelia lusitaniae*, *Borrelia spielmanii*, *Borrelia valaisiana*, and *R. raoultii*, all of which were *R. raoultii* strain Jongejan. In *H. concinna*, we found DNA of a *Babesia* sp., *Rickettsia helvetica*, and an organism closely related to *Theileria capreoli*. Lastly, *I. ricinus* was positive for *Anaplasma phagocytophilum*, *Borrelia afzelii*, *Borrelia burgdorferi* sensu stricto, *Borrelia garinii*/*Borrelia bavariensis*, *B. lusitaniae*, *B. spielmanii*, *B. valaisiana*, *Candidatus* Neoehrlichia mikurensis, *Rickettsia helvetica*, *Rickettsia monacensis*, and *Theileria* (*Babesia*) *microti* DNA. The detection of DNA of the *Babesia* sp. and an organism closely related to *Theileria capreoli*, both found in *H. concinna* ticks, is novel for Austria.

## 1. Introduction

In a previous study, we isolated and cultivated the novel *Rickettsia raoultii* strain Jongejan from Austrian *Dermacentor reticulatus* ticks [1]. *Rickettsia raoultii* belongs to the spotted fever group rickettsiae and can cause *Dermacentor*-borne necrotic erythema and lymphadenopathy (DEBONEL), tick-borne lymphadenopathy (TIBOLA), and scalp eschar and neck lymphadenopathy (SENLAT) in humans [2]. The main vectors of *R. raoultii* are *Dermacentor* ticks, specifically *D. reticulatus* and *D. marginatus* [3], but occasionally, (DNA of) *R. raoultii* is detected within other tick species, of which the vector capacity must still be investigated and confirmed [4,5].

Rickettsiae, including *R. raoultii,* are considered emerging tick-borne pathogens with their vector ticks expanding to novel loci worldwide, particularly in Europe [6,7]. Rickettsiosis is often an underdiagnosed disease, and many aspects are neglected [8]. With the isolation of a molecularly distinct *R. raoultii* strain, the question was raised whether this isolate is unique or whether this strain is more widely present within Austrian ticks. To assess this issue, a retrospective study was conducted to screen ticks collected from dogs in a previous study [9]. We chose to screen these ticks as this would enable us to cover a large area throughout most of the year. Additionally, to acquire a broad overview of tick-borne microbes present within the study area, we analyzed the collected ticks using the reverse line blot (RLB) assay. This enabled us to simultaneously screen for the presence of a large panel of tick-borne microorganisms, including *R. raoultii* strain Jongejan. Using this method, we obtained an extensive impression of tick-borne pathogens of the genera *Anaplasma*, *Babesia*, *Borrelia*, *Neoehrlichia, Rickettsia*, and *Theileria* present within the study area.

## 2. Materials and Methods

### 2.1. Ticks

**Figure 1:**
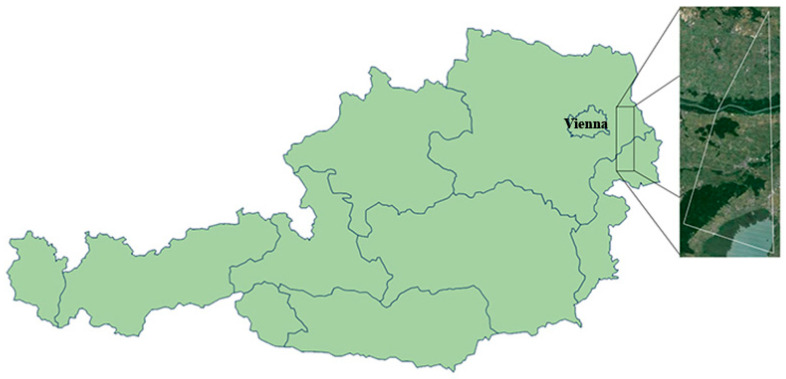
Map of Austria. The study site is shown in the satellite image excerpt, bordered in white. The map is modified from Google Maps.

The ticks were collected during a previous study conducted in 2008 [9]. Throughout that study, ticks were collected from dogs on a daily basis. The dogs were walked in an area east of Vienna, Austria (48°6′54″ N 16°42′9″ E; 48°17′56″ N 16°49′49″ E; 47°55′2″ N 16°36′50″ E; 47°51′51″ N 16°50′56″ E; https://www.google.com/maps/d/edit?mid=1ZT2orCQfXxZJUNUZmEaYWSMQIn5S9-7N&usp) (Figure 1). All ticks, in various stages of engorgement, were morphologically identified and stored individually at −20 °C until later use. As the molecular identification and/or confirmation of *Ixodes ricinus* ticks remained inconclusive, we refer to these ticks as part of the *I. ricinus* complex. Of these ticks, 75 (47 females and 28 males) *D. reticulatus*, 44 (21 females, 7 males, and 16 nymphs) *Haemaphysalis concinna,* and 55 (52 females and 3 males) ticks of the *I. ricinus* complex were randomly selected for molecular analysis.

### 2.2. DNA Extraction

DNA was extracted from the ticks by applying the Qiagen DNeasy Blood & Tissue Kit (Qiagen, Hilden, Germany). Briefly, individual ticks were cut in halves or quarters, depending on their size and using a sterile scalpel blade per individual tick, and homogenized in sterile 1.5 mL microcentrifuge tubes filled with 180 µL ATL buffer using disposable pestles (Bel-Art Products, VWR, Vienna, Austria). The lysis step was performed overnight at 56 °C. The remaining steps were carried out according to the manufacturer’s instructions. The DNA extractions obtained were stored at −20 °C until further use.

### 2.3. Reverse Line Blot-PCR

Several genus-specific RLB-PCRs were carried out as previously described [1,4] using the biotinylated primers listed in Table 1. The PCR reaction mix (25 μL total volume) contained 5 μL (5×) of Phire reaction buffer, 200 nmol/L of each dNTP (Solis BioDyne, Tartu, Estonia), 400 nmol/L of each primer per specific primer pair, 0.125 units of Phire Hot Start II DNA Polymerase (Thermo Scientific, Vienna, Austria), 2.5 μL of template DNA, and PCR-grade water (Sigma-Aldrich, Vienna, Austria). Several touchdown PCR protocols were used as described previously [1,4]. The resulting amplicons were analyzed using RLB hybridization as described previously [4].

### 2.4. Molecular Confirmation of Morphological Identification of Ixodes Ricinus

To confirm the morphological identification of *I. ricinus* complex ticks, we amplified part of the 16S rRNA gene as previously described [21,22] using the primers shown in Table 1. The resulting amplicons were sent to Microsynth (Balgach, Switzerland) for bidirectional sequencing. Morphological identification of *I. ricinus* complex ticks is difficult after the discovery of *Ixodes inopinatus* [23] due to the large similarities between these species and the fact that they inhabit the same geographical areas. The consensus sequences obtained were analyzed, and unique sequences were submitted to GenBank at the National Center for Biotechnology Information (NCBI) (http://www.ncbi.nlm.nih.gov/genbank/). The sequences can be accessed by using the accession numbers MW666053 and MW666054.

### 2.5. Sequencing

The RLB uses a set of 43 different oligonucleotide probes per membrane, upon which the PCR products are hybridized. This panel of oligonucleotide probes consists of a mixture of genus- and species-specific probes [4]. As there is limited space per membrane, we used the genus-specific probes as a backup should we fail to detect a species-specific signal. The genus-specific probes can be considered “catch-all” probes. When any of these catch-all signals were observed without a species-specific signal, we sequenced the RLB-PCR amplicon. Further sequencing was also used to confirm the presence of *R. raoultii* strain Jongejan. For this purpose, we sequenced the rickettsial *ompA*, *ompB*, *sca4*, *gltA*, and 16S rRNA genes and the 23S–5S rRNA intergenic spacer (IGS).

All PCR reactions for sequencing were conducted with nonbiotinylated primers (Table 1) and an increased polymerase concentration. The PCR mixtures (25 µL total volume) were identical as previously mentioned. However, we used 0.5 units instead of 0.125 units of Phire Hot Start II DNA Polymerase (Thermo Scientific, Vienna, Austria). The resulting PCR fragments were analyzed on agarose gels, excised, and purified by applying a gel purification kit (QIAquick Gel Extraction Kit, Qiagen, Hilden, Germany). Purified rickettsial PCR amplicons were sent to Microsynth (Balgach, Switzerland) for bidirectional sequencing. To obtain longer sequences, the purified “catch-all-only” RLB-PCR fragments were cloned into the cloning vector pJET1.2 according to the respective kit instructions (CloneJET PCR Cloning Kit, Thermo Fisher Scientific, Vienna, Austria). The obtained constructs were used to transform *Escherichia coli* DH5α cells. Plasmids were isolated from overnight cultures of the transformed *E. coli* cells using a plasmid purification kit (GeneJET Plasmid Miniprep Kit, Thermo Fisher Scientific, Vienna, Austria) as described in the manual. The purified plasmids were sent for bidirectional sequencing to Microsynth (Balgach, Switzerland) using the pJET1.2 forward and reverse sequencing primers.

The obtained sequences were truncated when required (plasmid and primer regions removed). The consensus sequences were analyzed, and unique sequences were submitted to GenBank (http://www.ncbi.nlm.nih.gov/genbank/), which are accessible using the accession numbers MW646028–MW646032.

## 3. Results

### 3.1. Identification of Ixodes Ticks by Sequencing

Sequencing part of the 16S rRNA gene resulted in two distinctive sequences (MW666053 and MW666054). Sequence MW666053 is 99.15% to 100% similar to *I. ricinus* (KM211785–KM211788) and 98.58% and 98.87% similar to *I. inopinatus* (KM211789 and KM211789, respectively). Sequence MW666054 is 98.31% to 98.87% similar to *I. ricinus* (KM211785–KM211788) and 99.44% similar to *I. inopinatus* (KM211789 and KM211789).

### 3.2. Molecular Detection of Tick-Borne Microorganisms

The DNA of a large variety of tick-borne microorganisms was detected using RLB and subsequent sequencing (Table 2). The most abundant organisms were of the genus *Rickettsia* with 16 positive ticks. Ten ticks were positive for *R. raoultii*, 5 ticks were positive for *R. helvetica*, and *R. monacensis* was detected in 1 tick. The second most frequently detected organisms belonged to the *Borrelia burgdorferi* sensu lato complex. A total of 13 ticks were positive, 4 ticks were positive with *B. valaisiana*, and 3 ticks with *B. lusitaniae*. *B. spielmanii* and *B. afzelii* were both detected in 2 ticks. Lastly, *B. burgdorferi* sensu stricto and *B. garinii/B. bavariensis* were detected in 1 tick (the methods applied failed to discriminate between *B. garinii* and *B. bavariensis*). The third most often identified genus was *Anaplasma* with 6 ticks positive for *A. phagocytophilum*. The fourth most frequently detected genus was *Babesia* in 4 ticks. Subsequent sequencing of the 18S rRNA gene fragment used for RLB revealed that all 4 ticks (MW646028–MW646031) were positive for a *Babesia* sp. closely related to *Babesia* spp. detected in the Russian regions Amur, Irkutsk, and Khabarovsk (99.72% to 100% similarity with KJ486560.1, KJ486562.1, KJ486563.1, KJ486564.1, KJ486566.1, KJ486567.1, and KJ486568.1). *Theileria* spp. DNA was present in 2 ticks, 1 tick was positive for *T.* (*Babesia*) *microti*, and additional sequencing revealed that the second tick (MW646032) was positive for DNA closely related to *T. capreoli* DNA (99.74% similarity with MH085202.1 and DQ866842.1) previously detected in Spain and China, respectively. Finally, a single tick was positive for *Candidatus* Neoehrlichia mikurensis.

### 3.3. Rickettsia raoultii Sequencing

Ten ticks tested positive for *R. raoultii* DNA by RLB (Table 2). To test which *R. raoultii* strain was present within these ticks, several genes were amplified and subsequently sequenced. After analysis, all *R. raoultii* sequences obtained were 100% identical to the *R. raoultii* strain Jongejan sequences, with a special emphasis on the *sca4* gene and the 23S–5S rRNA IGS. The *sca4* gene and the IGS region are most notably different between *R. raoultii* strain Jongejan and other *R. raoultii* strains. Specifically, the *sca4* gene turned into a pseudogene by a single nucleotide inclusion, and there was a 50-base pair deletion in the 23S–5S rRNA IGS.

## 4. Discussion and Conclusions

### 4.1. Tick Collecting

There are several methods for collecting ticks from the environment, including flagging/dragging [24], carbon dioxide trapping [25], and collecting ticks directly from the vegetation [1]. All these methods have their own benefits but are labor-intensive, especially when areas would be sampled every day. A study conducted in 2008 [9] showed that dogs prove to be a valuable resource for the collection of ticks in a large region with the possibility of obtaining ticks on a daily basis. Screening ticks collected from a host, it should be noted that the prevalence numbers of the observed microorganisms are biased. In general, it is impossible to determine whether the screened tick was positive for a specific pathogen prior to or following the blood meal during which the tick was collected. Additionally, infection of naïve ticks through cofeeding with positive ticks on the same host plays a significant role [26]. During cofeeding, microbes transmitted by positive ticks are able to infect naïve ticks without requiring the host animal to be (systemically) infected [26]. Therefore, overall tick-borne pathogen prevalence cannot be used for meaningful analysis. However, the presence of certain tick-borne microbes within the study area can still be confirmed this way.

### 4.2. Molecular Identification of Ixodes Ticks

The morphological identification of *I. ricinus* complex ticks is difficult, and molecular confirmation by sequencing the 16S rRNA gene is advisable. Estrada-Peña and colleagues [23] described the morphological characteristics and molecular sequences of the 16S rRNA gene belonging to both *I. ricinus* and *I. inopinatus*. The similarity between *I. ricinus* (KM211785–KM211788) and *I. inopinatus* (KM211789 and KM211789) 16S rRNA is 98.47% to 99.23%. As mentioned in the results section, our obtained sequence MW666053 is 99.15% to 100% similar to *I. ricinus* (KM211785–KM211788) and 98.58% and 98.87% similar to *I. inopinatus* (KM211789 and KM211789, respectively). Sequence MW666054 is 98.31% to 98.87% similar to *I. ricinus* (KM211785–KM211788) and 99.44% similar to *I. inopinatus* (KM211789 and KM211789). We therefore consider ticks with sequence MW666053 to belong to *I. ricinus* sensu stricto. However, ticks with the 16S rRNA gene sequence MW666054 are difficult to place as neither *I. ricinus* nor *I. inopinatus* 16S rRNA sequences are closely similar. We therefore refrain from precisely identifying our screened *I. ricinus* and rather refer to these ticks as belonging to the larger *I. ricinus* complex.

### 4.3. Tick-Borne Microorganisms Screening

Most of the detected microorganisms had previously been detected in Austria and the surrounding countries [4,27,28]. This proves that the applied approach for screening ticks collected from dogs is a useful method for screening for the occurrence of microbes in the environment.

*Anaplasma phagocytophilum* is usually detected in 0.7% to 33.9% of the questing ticks, with a highly diverse prevalence between countries [4,29,30,31]. Clinical cases are sporadically presented in Austria [32]. Spirochetes of the *B. burgdorferi* sensu lato complex are seen in ticks in similarly large varying numbers in Europe (i.e., between 8.6% and 25.6%) [4,33,34]. However, *Dermacentor* spp. and *Haemaphysalis* spp. ticks are not often linked to *B. burgdorferi* sensu lato spirochetes [35]. To the best of our knowledge, *H. concinna* is not known to be infected with *B. afzelii*. Regarding *D. reticulatus* and *D. marginatus*, only a few authors have described (the DNA of) *B. burgdorferi* sensu lato spirochetes in these ticks [36,37]. Therefore, identifying the DNA of these spirochetes in *D. reticulatus* ticks in this study is remarkable. Additionally, *B. lusitaniae* and *B. valaisiana* are mostly associated with (ticks collected from) lizards [38] or birds [39], respectively. This makes the detection of the DNA of these *Borrelia* spp. in *D. reticulatus* even more interesting, as both birds and reptiles are only rarely infested by *D. reticulatus* ticks [35]. However, as the ticks were collected from dogs, we cannot rule out that the dogs were positive but asymptomatic or whether only DNA was present within the ticks and no viable spirochetes. Nevertheless, our findings, in combination with the results of other studies, definitely validate additional research into the *B. burgdorferi* sensu lato vector capacity by *Dermacentor* ticks. *Candidatus* Neoehrlichia mikurensis is a relatively newly discovered human pathogen that was recently cultivated for the first time [40]. This pathogen is detected in ticks at 0.5% to 4.3% [4,41] and in some locations at even up to 25.5% [42]. Detection of *Theileria* (*Babesia*) *microti* in *Ixodes* ticks is of high medical relevance, especially as *T.* (*Babesia*) *microti* is the major causative agent of human babesiosis in Europe alongside *Babesia divergens* and *Babesia venatorum* [43]. This fact is of particular importance for blood banks. Blood from chronically infected asymptomatic blood donors may infect persons if not thoroughly screened for the presence of piroplasms prior to blood transfusion [44]. Infection with *T.* (*Babesia*) *microti* through blood transmission can cause severe complications and death in about a fifth of the cases [44]. Because of its rare occurrence, babesiosis as an infection is often not considered and thus underdiagnosed. *Theileria* (*Babesia*) *microti* is an organism that does not fit to the *Babesia* or *Theileria* genera on a molecular basis (18S rRNA) [45]. Additionally, *T.* (*Babesia*) *microti* is not transovarially transmittable, and the occurrence of schizogony in lymphocytes has been observed. Therefore, the pathogen cannot be classified as a *Babesia* sp. and, for the time being, is instead classified as a *Theileria* sp. [45]. Rickettsiae are among the most prevalent microorganisms in ticks, including the tick species studied in this paper, and often are considered tick endosymbionts [46]. Prevalence percentages ranging between 16.8% and 33.7% for rickettsiae in ticks are not uncommon [4,41,47], and some studies have even shown higher rates of up to 58% infected ticks [48].

Lastly and most importantly, throughout the previous study in 2008 [9], no symptoms of tick-borne diseases were witnessed in the dogs that participated.

### 4.4. Rickettsia raoultii Strain Jongejan

One of the reasons that the current study was carried out was to see whether the *R. raoultii* strain Jongejan that we isolated in 2016 [1] was unique or more widely present in Austria. To this end, we sequenced the *ompA*, *ompB*, *sca4*, *gltA*, and 16S rRNA genes and the 23S–5S rRNA IGS region of all *R. raoultii*-positive ticks. Sequencing revealed that all *R. raoultii* sequences obtained were 100% identical to the previously isolated and characterized *R. raoultii* strain (KX500092–KX500097). Thus, we were able to confirm that *R. raoultii* strain Jongejan was widely present in the study area in as early as 2008. Further research is required to determine whether *R. raoultii* strain Jongejan possesses additional molecular alterations and perhaps fits into an evolutionary niche, especially as it was unexpected to find no other strain than *R. raoultii* strain Jongejan in this region.

### 4.5. Theileria capreoli

In the course of this study, we detected a *Theileria* sp. closely related to *T. capreoli in* a *H. concinna* nymph. The sequences found were 99.74% similar to *T. capreoli* sequences observed in the Heilongjiang Province, northeastern China (MH085202.1), and in northern Spain (DQ866842.1). The vector tick has not yet been determined for this *Theileria* sp. Interestingly, in Hungary, stable flies (*Stomoxys calcitrans*) showed positivity for *T. capreoli* DNA without reservoir animals (e.g., cervids) being in close proximity [49].

In several studies, wild cervids and bovids were identified carriers of *T. capreoli*. Among these cervids were fallow deer (*Dama dama*), red deer (*Cervus elaphus*), roe deer (*Capreolus capreolus*), Siberian roe deer (*Capreolus pygargus*), and bovids such as mouflon (*Ovis gmelini*) [50,51,52]. An interesting exception to these findings was shown in a paper describing a study in Croatia, in which *T. capreoli* was detected in gray wolves (*Canis lupus*) [53]. This suggests that *T. capreoli* infections may not be limited to cervids only. Furthermore, if wolves can become positive, the question of whether dogs may become asymptomatic carriers as well arises. Looking further into this, we identified one study in which the researchers had detected the DNA of *T. capreoli* in the myocardial tissue of a single dog during postmortem diagnostic screening [54]. (The dog died from septic shock complicated by disseminated intravascular coagulation.) This could shed new light on the Hungarian findings of positive stable flies in the absence of natural hosts and the presence of dogs, especially as stable flies are known to bite dogs when they are in close proximity to the stables [55].

### 4.6. Babesia spp.

Our findings also include the detection of babesial sequences closely related to *Babesia* spp. detected in a Russian study in the Amur, Irkutsk, and Khabarovsk regions (99.72% to 100% similarity with KJ486560.1, KJ486562.1, KJ486563.1, KJ486564.1, KJ486566.1, KJ486567.1, and KJ486568.1). Further studies are required to investigate the specific *Babesia* spp. and the impact they have on human and wildlife health, especially as new *Babesia* spp. are frequently discovered and the incidence of human babesiosis cases has increased significantly over the past decade [56]. *Haemaphysalis concinna* is a suspected vector of piroplasms [57,58], the primary hosts of which have been identified in several studies as small- and medium-sized mammals and birds [59,60,61,62]. However, *H. concinna* of all life stages can also be found in larger animals, such as wild and domesticated ungulates [63], and carnivores such as foxes and dogs [60,64]. Humans are occasionally bitten by *H. concinna* as well [27], making *H. concinna* a tick species of high medical importance that requires further study.

## Figures and Tables

**Table 1:** Primers used for the reverse line blot and sequencing.

Target Organisms	Sequence (5′–3′)	Target Region	Reference
RLB primers *			
*Anaplasma*/*Ehrlichia* spp.	GGAATTCAGAGTTGGATCMTGGYTCAG	16S ribosomal RNA gene	[10]
	(Biotin-)CGGGATCCCGAGTTTGCCGGGACTTYTTCT		[11]
*Babesia*/*Theileria* spp.	GACACAGGGAGGTAGTGACAAG	18S ribosomal RNA gene	[12]
	(Biotin-)CTAAGAATTTCACCTCTGACAGT		[12]
*Borrelia burgdorferi* sensu lato	ACCATAGACTCTTATTACTTTGACCA	5S–23S ribosomal RNA intergenic spacer	[13]
	(Biotin-)GAGAGTAGGTTATTGCCAGGG		[13]
*Rickettsia* spp.	GAACGCTATCGGTATGCTTAACACA	16S ribosomal RNA gene	[14,15]
	(Biotin-)CATCACTCACTCGGTATTGCTGGA		[14,15]
	GATAGGTCRGRTGTGGAAGCAC	23S–5S ribosomal RNA intergenic spacer	[16]
	(Biotin-)TCGGGAYGGGATCGTGTGTTTC		[16]
**Additional sequencing primers**			
*Rickettsia* spp.	ATGAGTAAAGACGGTAACCT	*sca4*	[17]
	AAGCTATTGCGTCATCTCCG		[17]
	ATGGCGAATATTTCTCCAAAA	*ompA*	[18]
	GTTCCGTTAATGGCAGCATCT		[18]
	AAACAATAATCAAGGTACTGT	*ompB*	[19]
	TACTTCCGGTTACAGCAAAGT		[19]
	GCAAGTATCGGTGAGGATGTAAT	*gltA*	[20]
	GCTTCCTTAAAATTCAATAAATCAGGAT		[20]
Molecular identification of ticks	CTGCTCAATGATTTTTTAAATTGCTGTGG	16S ribosomal RNA gene	[21]
	CCGGTCTGAACTAGATCAAGT		[22]

* The RLB-PCRs were conducted with biotinylated reverse primers. Nonbiotinylated primers were used for sequencing.

**Table 2:** Tick-borne microorganism DNA detected in ticks collected from dogs.

	*Dermacentor reticulatus*			*Haemaphysalis concinna*				*Ixodes ricinus*		
	Tot. (*n* = 75)	Female (*n* = 47)	Male (*n* = 28)	Tot. (*n* = 44)	Female (*n* = 21)	Male (*n* = 7)	Nymphs (*n* = 16)	Tot. (*n* = 75)	Female (*n* = 47)	Male (*n* = 28)
*Anaplasma phagocytophilum*	**5**	1	4					**1**	1	
*Babesia* spp.				**4**		2	2			
*Borrelia afzelii*				**1**			1	**1**	1	
*Borrelia burgdorferi* sensu stricto								**1**	1	
*Borrelia garinii/Borrelia bavariensis*								**1**	1	
*Borrelia lusitaniae*	**2**		2					**1**	1	
*Borrelia spielmanii*	**1**	1						**1**	1	
*Borrelia valaisiana*	**3**	2	1					**1**	1	
*Ca*. Neoehrlichia mikurensis								**1**		1
*Rickettsia helvetica*				**2**		1	1	**3**	2	1
*Rickettsia monacensis*								**1**	1	
*Rickettsia raoultii*	**10**	1	9							
*Theileria capreoli*				**1**			1			
*Theileria* (*Babesia*) *microti*								**1**	1	

## Data Availability

Unique sequences have been deposited at NCBI’s GenBank and are available using the accession numbers MW646028–MW646032, MW666053, and MW666054. Other data are available upon request.
